# A New Multichannel Parallel Network Framework for the Special Structure of Multilead ECG

**DOI:** 10.1155/2020/8889483

**Published:** 2020-12-03

**Authors:** Peng Lu, Hao Xi, Bing Zhou, Hongpo Zhang, Yusong Lin, Liwei Chen, Yang Gao, Yabin Zhang, Yanhua Hu

**Affiliations:** ^1^Department of Automation, School of Electrical Engineering, Zhengzhou University, Zhengzhou 450001, China; ^2^Collaborative Innovation Center of Internet Healthcare, Zhengzhou 450052, China; ^3^School of Information Engineering, Zhengzhou University, Zhengzhou 450001, China; ^4^State Key Laboratory of Mathematical Engineering and Advanced Computing, Zhengzhou 450001, China; ^5^School of Software, Zhengzhou University, Zhengzhou 450002, China

## Abstract

Electrocardiogram (ECG) contains the rhythmic features of continuous heartbeat and morphological features of ECG waveforms and varies among different diseases. Based on ECG signal features, we propose a combination of multiple neural networks, the multichannel parallel neural network (MLCNN-BiLSTM), to explore feature information contained in ECG. The MLCNN channel is used in extracting the morphological features of ECG waveforms. Compared with traditional convolutional neural network (CNN), the MLCNN can accurately extract strong relevant information on multilead ECG while ignoring irrelevant information. It is suitable for the special structures of multilead ECG. The Bidirectional Long Short-Term Memory (BiLSTM) channel is used in extracting the rhythmic features of ECG continuous heartbeat. Finally, by initializing the core threshold parameters and using the backpropagation algorithm to update automatically, the weighted fusion of the temporal-spatial features extracted from multiple channels in parallel is used in exploring the sensitivity of different cardiovascular diseases to morphological and rhythmic features. Experimental results show that the accuracy rate of multiple cardiovascular diseases is 87.81%, sensitivity is 86.00%, and specificity is 87.76%. We proposed the MLCNN-BiLSTM neural network that can be used as the first-round screening tool for clinical diagnosis of ECG.

## 1. Introduction

ECG signal is a periodic and nonstationary time series biological signal. It is based on the principle that cardiac electrical activity presents a complex nonlinear dynamic system [1]. ECG contains the rhythmic features of continuous heartbeat and morphological features of ECG waveforms [2]. The nonlinearity and complexity of an abnormal ECG make the waveform of a heart disease unique, and differences are observed in time series [3].

Using prior knowledge and deep learning are the two main technical routes of ECG automatic analysis for diagnosis [4, 5]. An auxiliary diagnosis based on prior knowledge relies on manually extracted ECG features. Differences among ECG lead to the uncertainty of feature extraction and reduce the classification accuracy of an algorithm. Auxiliary diagnosis based on deep learning is data-oriented and can automatically extract ECG features through a model, mine complex association patterns and rich information from data, effectively prevent uncertainty in manual feature extraction, and provide a feasible technical way for ECG big data analysis. For cardiovascular disease diagnosis, differences among ECG are usually addressed by using deep learning algorithms in mining ECG morphological and rhythmic features [5]. In Fan et al.'s work [6], a multiscale fused convolutional neural network (MS-CNN) is designed to solve the problem of short single-lead ECG signal atrial fibrillation detection. The network extracts the features of ECG records from different times by establishing two CNNs with different convolution kernel sizes. Hannun et al. [7] used an original ECG time series as input with a 34-layer CNN and generated a prediction tag once per second; their experimental results are better than those of cardiologists in terms of sensitivity and accuracy. Tan et al. [8] implemented a Long Short-Term Memory (LSTM) neural network on ECG to diagnose coronary artery disease; in this method, 5 s ECG signals were cut into short sequences and convoluted for the reduction of data points; then, LSTM was applied to a time feature extracted from the convolution sequence; their model achieved a diagnostic accuracy of 99.85%. Singh et al. [9] used the recurrent neural network (RNN), gating recursive unit (GRU), and LSTM to classify heartbeats and concluded that LSTM was better than RNN and GRU in detecting arrhythmia.

Thus, most ECG classification models based on deep learning focus on the classification of single-lead ECG beats. Decreasing the amount of information may be computationally friendly and results in good performance in some cases. However, from a clinical point of view, cardiologists usually make diagnosis on the basis of 12-lead data [10], which they use in evaluating as much information as possible before drawing a final diagnosis conclusion. Therefore, studying 12-lead long-term data is extremely important. The above work has proven the application of many widely used deep learning networks in ECG recognition, but studies of the multilead ECG, which have the special structures of physiological signal-customized deep learning modes, are few. The following points should be considered. First, CNN was originally designed for image recognition. Multilead ECG data have no correlation to one another at different times and lead, and thus considering that these data are two-dimensional digital images and classifying them through CNN are inappropriate. Second, CNNs are suitable to obtaining spatial features, whereas LSTM are effective in learning temporal features. Different types of classifiers have different modes of feature expression for the same data [11]. The correct combination of different classifiers and the full extraction of rhythmic and morphological features in ECG enhances performance. Third, the sensitivity of different cardiovascular diseases to morphology and rhythm varies [12]. For example, in the diagnosis of myocardial infarction, changes in QRS complex, P wave morphology, and ST-T segment are considered [13]. By contrast, change in rhythm is essential to the diagnosis of tachycardia [14]. The challenge of using the sensitivity of disease to a feature is considered as well.

In this study, based on the above viewpoints, we propose a combination of multiple neural networks, the multichannel parallel neural network model (MLCNN-BiLSTM), and use it in the multiclass classification disease recognition of multilead ECG. In summary, the main contributions can be described as follows:

1. According to the special structure of a multilead ECG, a novel MLCNN structure is proposed. This structure can effectively extract the strong correlation information of sample points in multilead ECG while ignoring irrelevant information.
2. The multilead ECG is modeled on the basis of multiple neural networks and multichannel parallel. On the one hand, channel 1 uses a MLCNN to extract the morphological features of multilead ECG data. On the other hand, channel 2 uses a BiLSTM to extract the rhythmic characteristics of lead II ECG data.
3. Core threshold parameters are initialized and the backpropagation algorithm is used to update automatically to control the weight ratio of high-dimensional time-space features extracted by multichannel parallel extraction in weighted fusion. The sensitivity of different cardiovascular diseases to morphology and rhythm is automatically learned, and the multiclass classification auxiliary diagnosis model is established.

The experimental results show that the proposed combination between multiple neural networks and multichannel parallel neural network (MLCNN-BiLSTM) achieves good results in multilead ECG classification. The effectiveness of disease screening is verified through visual analysis of the high-dimensional features of the model after time-space fusion.

## 2. Methods

### 2.1. Problem Description

Cardiovascular disease recognition can be transformed into a multiclass classification task, and its ultimate goal is to synthesize a highly complex nonlinear decision function. As shown in equation (1). The input sequence is a set of ECG signals, *X*=[*x*1, *x*2, *x*3,…, ]. The output is the corresponding label *R*=[*r*1, *r*2, *r*3,…, ]. Each output label corresponds to the category of an input ECG. By taking cross entropy as the loss function, a highly complex nonlinear decision function is fitted by all the samples in the training set and optimized for minimal loss value:

(1) $$\begin{array}{c} \text{Loss}\left(X,r\right)=-\log\left(\frac{\exp\left(p\left(X,r\right)\right)}{\sum_{j}\exp\left(p\left(X,j\right)\right)}\right), \end{array}$$

where *p*(*X*, *j*) is the probability that the model predicts as label *j* for input *x* and *r* is the real label.

### 2.2. MLCNN

**Figure 1:**
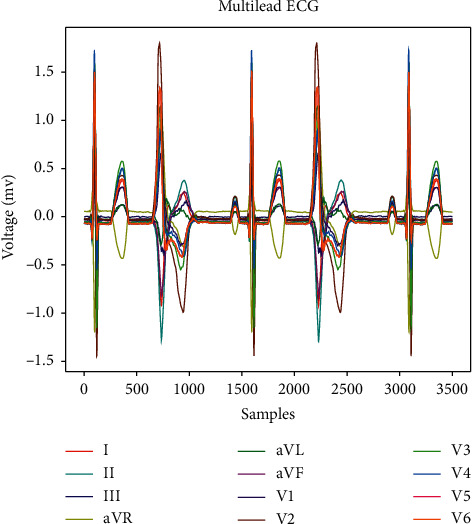
Correlation of 12-lead ECG.

Reference [15] points out that there is a significant correlation between the data of different leads at the same time in the standard 12-lead ECG signal. As shown in Figure 1, by plotting the data of different leads in the same coordinate system, the correlation between different leads of the multilead ECG can be observed intuitively. For each single-lead ECG signal, data at different times have a time series correlation. We think that the data of different leads at different times are irrelevant.

**Figure 2:**
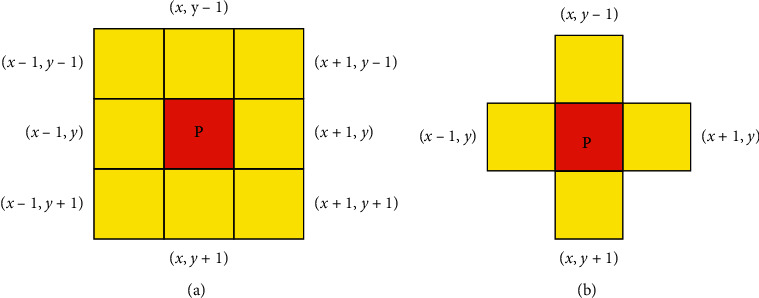
(a) 8-neighborhood structure (N8(*p*)) of each pixel in 2D digital image; (b) 4-neighborhood structure (N4(*p*)) of each sampling point in multilead ECG.

A 2D convolutional neural network (2D-CNN) uses two-dimensional convolution operation, which is originally designed for the recognition of two-dimensional digital images [16]. However, owing to the particularity of multilead ECG structures, considering that it is a two-dimensional digital image and using CNN to classify it are inappropriate. In a two-dimensional digital image, for each pixel *p*(*x*, *y*), adjacent to (*x* − 1, *y* − 1), (*x*, *y* − 1), (*x*+1, *y* − 1), (*x* − 1, *y*), (*x*+1, *y*), (*x* − 1, *y*+1), (*x*, *y*+1), and (*x*+1, *y*+1), eight pixels are connected. It is called the eight-neighborhood structure [17], expressed by N8(*p*). Multilead ECG can be considered a four-neighborhood structure, expressed by N4(*p*). In each sampling point *P*(*x*, *y*), it is the same as the sampling points (*x*, *y* − 1) and (*x*, *y*+1) on different leads at the same time, and the sampling points at different times on the same lead (*x* − 1, *y*) and (*x*+1, *y*) have a strong correlation. The sampling points in the neighborhood of N8(*p*)-N4(*p*) have no correlation. As shown in Figure 2, the relevant neighborhood information of image pixels and multilead ECG sampling points is displayed.

In CNN, convolution is an effective way for extracting local region features. As shown in equations (2) and (3), for the calculation of the output feature map *Y*^*p*^, convolution kernel *W*^*p*,1^, *W*^*p*,2^,…, *W*^*p*,*D*^ convolves the input feature map. Then, the convolution results and a scalar bias are added for the production of the net input *Z*^*P*^ of the convolution layer. Finally, the output feature map *Y*^*p*^ is obtained by nonlinear activation function:

(2) $$\begin{array}{c} Z^{p}=W^{p}⊗X+b^{p}=\sum_{d=1}^{D}W^{p,d}⊗X^{d}+b^{p}, \end{array}$$

(3) $$\begin{array}{c} Y^{p}=\;f\left(Z^{p}\right), \end{array}$$

where *x* is the three-dimensional convolution kernel *W*^*p*^ ∈ *ℝ*^*m*×*n*×*D*^ (for multilead ECG structure, *D*=1), ⊗ is the convolution operation, and *f*(*·*) is a nonlinear activation function.

In the convolution operation of the 2D-CNN layer, each convolution kernel extracts the eight-neighborhood structure features of a digital image. When 2D-CNN is used to convolute multilead ECG, the feature information of sampling points in the neighborhood of N8(*p*)-N4(*p*) is extracted in each convolution process. As algorithm complexity increases, the high-dimensional feature expression of a feature map is combined with some irrelevant features after convolution. This effect introduces unnecessary feature information to deeper convolution operations. Given the special structure of multilead ECG and the deficiency of traditional 2D-CNN in processing multilead ECG, we design an MLCNN neural network for multilead ECG data.

**Figure 3:**
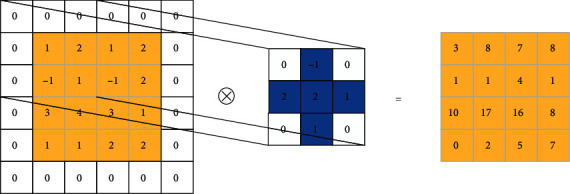
Convolution operation of MLCNN neural network.

The MLCNN, similar to traditional CNN, is composed of convolution and pooling layers. In the convolution layer of the MLCNN, we change the initialization state of each square convolution kernel; that is, only the weights on the N4(*p*) neighborhood of its central point are reserved, and the weights of the other central points on the non-N4(*p*) neighborhood are set to 0. The convolution operation is shown in Figure 3.

By setting the weight of the non-N4(*p*) neighborhood of the center point of the convolution kernel to 0, each convolution operation can accurately extract local features from the receptive field on the same lead at different times and different leads at the same time. Therefore, the unnecessary data features of different leads at different times are removed. This setting can reduce the number of times of convolution weighted summation by the convolution kernel, reduce the complexity of an algorithm, and have a positive effect on the fast convergence of a network.

### 2.3. Network Architecture

**Figure 4:**
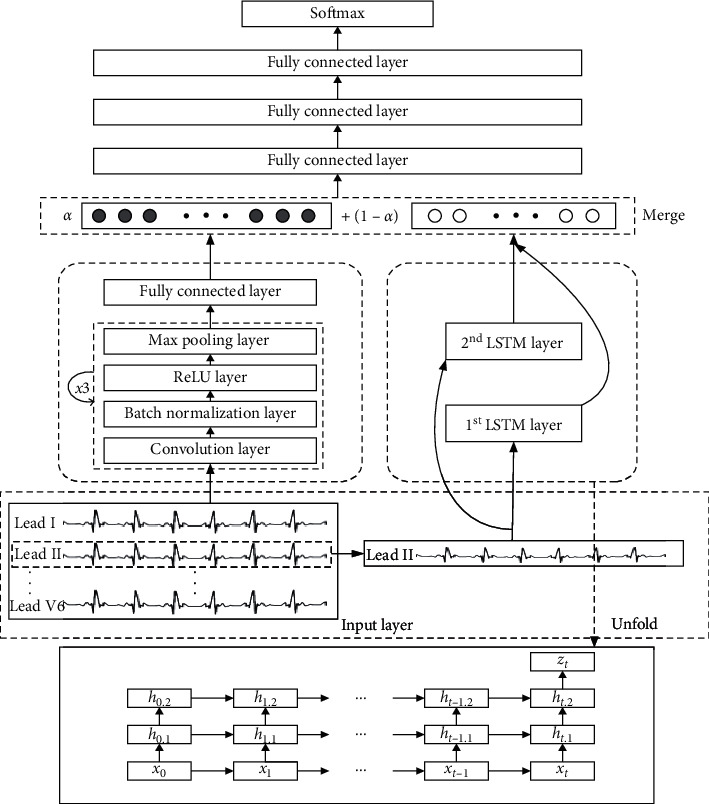
An illustration of the proposed MLCNN-BiLSTM architecture.

MLCNN can effectively extract the spatial morphological features of ECG. To explore the rhythmic features contained in ECG and improve the accuracy of classification and recognition, we propose a combination of multiple neural networks and multichannel parallel network model (MLCNN-BiLSTM). The structure is shown in Figure 4. The preprocessed 12-lead ECG data with a duration of 10 seconds are inputted into the input layer of MLCNN channel as a two-dimensional array (the size of the input is 12 ∗ 1900; we removed data from the first 50 and last 50 samples of each lead separately to eliminate data errors caused by manual manipulation during data collection) for the extraction of the morphological features of ECG waveforms. Only lead II data are selected from the complete data and inputted into the input layer of BiLSTM channel as a one-dimensional array (the size of the input is 1 ∗ 1900) for the extraction of the rhythmic characteristics of ECG signals. Lead II data contains abundant ECG features [18]. The two channels are carried out in parallel for the extraction of the high-dimensional and deep-level features of ECG. By initializing the core threshold weight parameters and using the backpropagation algorithm to update automatically, the high-dimensional spatial-temporal features extracted from the two channels are weighted and fused. The detection rate of disease classification is improved by controlling the weight output proportion. Finally, the softmax activation function is used for multiclass classification.

#### 2.3.1. MLCNN Channel

The channel information-sharing mechanism in the convolutional layer is conducive to the spatial fusion of different pieces of lead information [19]. The MLCNN channel is composed of three convolution blocks and a full-connection layer in a series. The convolution block is composed of an MLCNN convolution, batch normalization (BN), ReLU, and maximum pooling layers. For the arrangement of convolution blocks, we refer to the practice in [20]. This structure can extract features and good interpretability. In the convolution layer, features and their relative weights are extracted by a local connection. A backpropagation error algorithm is used to train the weight of each layer of each parameter in the convolution kernel.

The introduction of BN layer [21] solves the problem of internal covariate migration. Its advantage is that it allows a model to use a high learning rate in the training process and further reduces the overfitting of the model. Then ReLU linear rectification function is used to activate features extracted by the convolution layer. The calculation of nonlinear activation function is shown in the following equation:

(4) $$\begin{array}{c} y_{i}^{k}=\sigma \left(w^{k}x_{i}+b^{k}\right), \end{array}$$

where *W*^*k*^ and *b*^*k*^ are the weight and offset of the *k*-th convolution feature map, respectively, *x*_*i*_ is the input of the *k*-th convolutional layer, and *y*_*i*_^*k*^ is the output of the *k*-th convolutional layer.

In the proposed model, the maximum pooling layer is used to reduce the dimension of the feature vector of the convolution layer through subsampling, which reduces the complexity of subsequent calculation and can introduce translation invariance into a model. The output of the pooling layer with strides of two is half of the input length. The introduction of a full-connection layer ensures the consistency of the MLCNN channel output dimension and BiLSTM channel output dimension.

#### 2.3.2. BiLSTM Channel

**Figure 5:**
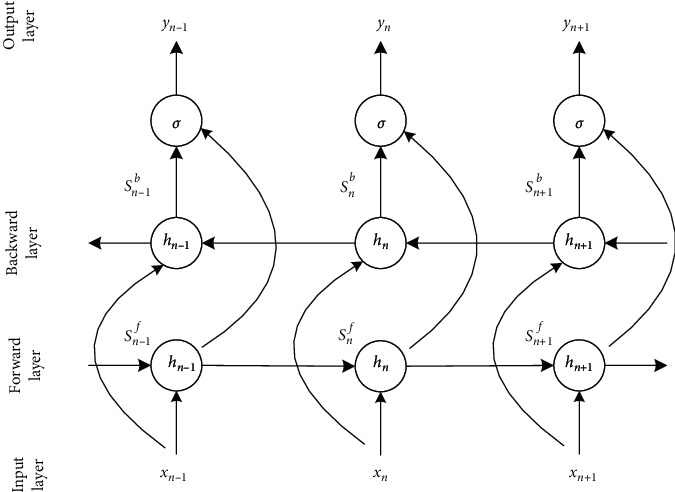
The BiLSTM neural network cell structure.

As the only lead of a single-lead ECG device, the standard lead II data contains rich heartbeat information. The data in the lead is time related. The output of a moment is related not only to the information of the past moment but also to the information of subsequent moments. The BiLSTM network uses two separate hidden layers to process data in two directions and then feeds the data to the same output layer. Features can be effectively extracted from time dimension [22]. As shown in Figure 5 and equation (5), the output *y*_*n*_ is a function of forward passing *s*_*n*_^*f*^, backward passing *s*_*n*_^*b*^, and the hidden state of corresponding weights and deviations.

(5) $$\begin{array}{c} y_{n}=\sigma \left(w_{f}s_{n}^{f}+w_{b}s_{n}^{b}+b_{h}\right), \end{array}$$

where *w*_*f*_ and *w*_*b*_ are forward weight and backward weight, *b*_*n*_ is deviation, and *σ* is softmax function.

#### 2.3.3. Channel Feature Fusion

For the exploration of the sensitivity of different cardiovascular diseases to morphology and rhythm, the core threshold weight parameter *α* is initialized and automatically updated through neural network learning. The parameter *α* controls the weight ratios of high-dimensional features from two channels for time-space fusion. Parameter *α* is a variable parameter that can be learned rather than a hyperparameter. We just initialize the parameter *α*. Here, the feature fusion method is not simple vector splicing but a pointwise addition. For MLCNN channel output space feature vector *v*_1_ ∈ *R*^*n*^, BiLSTM channel output time series feature vector *v*_2_ ∈ *R*^*n*^ is added to corresponding elements, as shown in the following equation:

(6) $$\begin{array}{c} v=\alpha \cdot v_{1}+\left(1-\alpha \right)\cdot v_{2}, \end{array}$$

where *v*={*x*_*i*_*|x*_*i*_=*v*_1_[*i*]+*v*_2_[*i*],  *i*=1,…, *n*} and *α* is the threshold weight.

## 3. Experiment

### 3.1. Data Set

We will use the data of Chinese Cardiovascular Disease Database (CCDD) [23] to study our proposed algorithm. All the data in the CCDD are from clinical practice. The standard 12-lead data with sampling rate of 500 Hz are used in actual collection. Data are manually annotated by many authoritative cardiovascular experts. Compared with other ECG experimental databases (such as MIT database), the CCDD database is more in line with clinical cardiovascular disease recognition algorithm research.

In the deep learning experiment in this paper, we select the standard 12-lead data of atrial arrhythmia, ventricular arrhythmia, myocardial infarction, ventricular hypertrophy, atrial hypertrophy, and normal heart rhythm from the CCDD, that is, leads I, II, III, AVL, AVF, AVR, and V1–V6, respectively, as the deep learning training data set. Ten seconds is the typical duration of ECG signal collection [24].

The total number of clinical ECG samples used in the experiment is 143,092, of which 108,120 are normal heart rate ECG samples and 34,972 are abnormal ECG samples. After disordering the ECG samples in each category, the training, verification, and test sets are divided at a ratio of 8 : 1 : 1. The training set has 114,472 ECG samples, the verification set has 14,310, and the test set has 14,310. The specific data distribution is shown in Table 1.

### 3.2. Preprocessing

The overall frequency range of the ECG waveform is 1–40 Hz. According to Nyquist sampling law, when the sampling frequency *fs*.max is greater than twice the highest frequency *f*max in a signal (*fs*.max > 2*f*max), a sampled digital signal completely retains the information of an original signal. Mesin [25] proposed an adaptive algorithm for the nonuniform downsampling of data used to reduce the mean sampling frequency of ECG data. It outperformed both uniform downsampling and compressive sensing in terms of the recovery of high amplitude or energetic components. The algorithm showed good accuracy in the identification of the heartbeats from downsampled data. Notice, for example, that the mean accuracy in identifying the heartbeats was over 98%. To reduce the number of sampling points and the complexity of subsequent calculations, we refer to the practice in [25] and then downsample the 10 s continuous ECG signal sampling rate from 500 Hz to 200 Hz.

The CCDD is a clinical ECG database, and its ECG data is directly collected from clinical patients, collated, and stored. The integrity of ECG data collected in the clinic is ensured. However, noises are present. Noise removal is conducive to deep learning for feature extraction and classification accuracy [26]. Three kinds of noise are present in an ECG signal, namely, baseline drift, power frequency interference, and EMG interference. Median filter, digital low-pass filter, and digital high-pass filter are often used to remove high-frequency and low-frequency noise from signals such as baseline drift and power frequency interference noise [27], and good results have been achieved. Conventional filtering methods for suppressing EMG interference cause *R*-peak clipping and the distortion of QRS complex wave in an ECG signal [28].

We first preliminarily processed the original ECG signal based on a median filter to remove baseline drift interference and then based on an FIR low-pass filter to remove high-frequency noise and power frequency interference and a FIR high-pass filter to remove low-frequency noise interference.  Algorithm 1 shows the process of preliminary processing.

Through the experiment, the median filter parameter *α*_median_ is 0.9, the FIR low-pass filter cut-off frequency *f*_clp_ is 40, the high-pass filter cut-off frequency is *f*_chp_ 2, and the filter order *F*_order_ is 8.

In order to avoid the influence of the subsequent process of removing EMG interference noise on the QRS complex, we need to identify the QRS complex in the preliminarily preprocessed ECG signal *y*_*f*_. The signal of the corresponding region is retained so that it will not be affected when the EMG interference noise is removed. We adopt the *R*-peak detection algorithm based on wavelet coefficients in [29]. The Haar wavelet basis is used to decompose the ECG signal into 5-layer discrete wavelet, and the fourth- and fifth-layer wavelet detail coefficients are selected to reconstruct the *R*-peak detection sequence; the amplitude threshold is set to search the position of the *R*-peak.  Algorithm 2 shows the *R*-peak detection algorithm.

**Figure 6:**
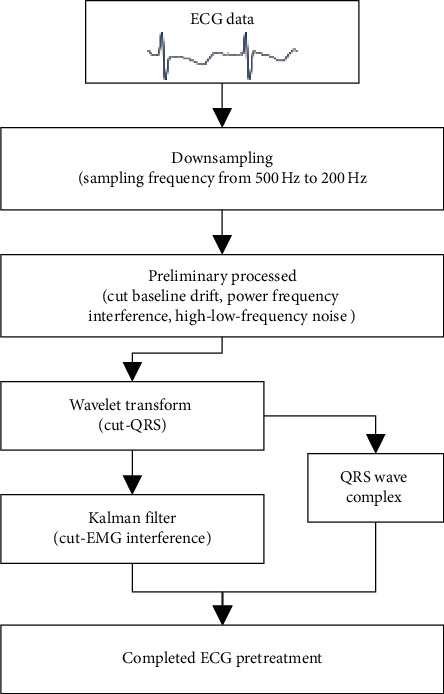
The framework of the data preprocessing algorithm.

We centered on the *R*-peak and searched forward/backward for the extreme point with the lowest amplitude, denoted as *Q*_loc_ and *S*_loc_, respectively. The region of the ECG signal where the sample point index values range between *Q*_loc_ and *S*_loc_ is the QRS complex wave region that should be preserved. Set the signal amplitude of this area to 0, and then remove the EMG interference signal in the ECG signal based on the Kalman filtering method. Restore the QRS complex area set to 0, and finally obtain the target ECG signal that removes myoelectric interference and retains the QRS complex shape. The complete flow chart of the preprocessing algorithm is shown in Figure 6.

**Figure 7:**
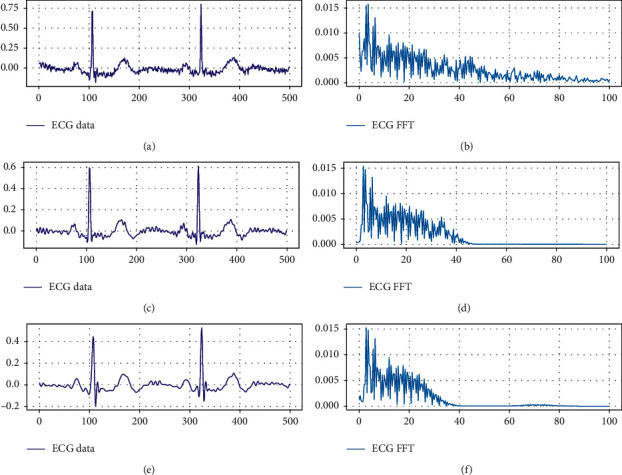
The ECG signal with and without noise removal. ((a) and (b)) Original ECG signal and spectrum map. ((c) and (d)) ECG signal and spectrum map after preliminary processing. ((e) and (f)) ECG signal and spectrum map without noise.

Figure 7 shows the results of each preprocessing step. It can be found that, after preliminary processing, the noise in the original ECG signal is suppressed to some extent, but there is still obvious EMG interference. After Kalman filtering, the ECG signal, especially the EMG noise between the two QRS conforming waves, is effectively suppressed.

### 3.3. Training Setup

The network parameters (weights and deviations) for optimal performance in a given task are considered nonconvex optimization problems [22]. In the multichannel parallel network model mentioned above, cross entropy is used as the cost function in this study:

(7) $$\begin{array}{c} J\left(\theta \right)=-\frac{1}{m}\sum_{i=1}^{m}y^{\left(i\right)}\log\left(h_{\theta }\left(x^{\left(i\right)}\right)\right)+\left(1-y^{\left(i\right)}\right)\log\left(1-h_{\theta }\left(x^{\left(i\right)}\right)\right), \end{array}$$

where *x*^(*i*)^ is the input ECG signal, *y*^(*i*)^ is the output category, *h*_*θ*_(*·*) is the activation function of input *x*^(*i*)^, and *m* is the number of training samples.

Algorithm 3 shows the whole process of model training.

Table 2 shows the detailed parameters of each layer of the proposed network model.

## 4. Result

We use three indicators, namely, accuracy (ACC), sensitivity (SE), and specificity (SP), to evaluate the performance of the proposed network. The ACC is the ratio of the sum of true positive and true negative to the total number of samples and reflects consistency between test and actual results. The SE (also known as recall, RE) is the proportion of true positive data correctly predicted to be positive. The higher the SE is, the greater the proportion of correct prediction is. SP, also known as negative predictive rate, is the ratio between true negative data correctly predicted to be negative and all actual negative samples. Three evaluation indicators can be calculated as follows:

(8) $$\begin{array}{c} \text{ACC}\left(\text{accuracy}\right)=\frac{\text{TPs}+\text{TNs}}{\text{TPs}+\text{TNs}+\text{FPs}+\text{FNs}},\\ \text{SE}\left(\text{sensitivity}\right)=\frac{\text{TPs}}{\text{TPs}+\text{FNs}},\\ \text{SP}\left(\text{specificity}\right)=\frac{\text{TNs}}{\text{TNs}+\text{FPs}}, \end{array}$$

where TP (true positive) represents the number of samples belonging to this class and classified into this class by the classifier. FP (false positive) represents the number of samples that do not belong to this class but are wrongly assigned to this class by the classifier. FN (false negative) represents the number of samples belonging to this class but assigned to other classes by the classifier. TN (true negative) represents the number of samples that do not belong to this class and have not been classified into this class by the classifier.

To make the deep learning model reach the optimal structure, a quantitative research method is used in determining the influences of various structures and network parameters on the experimental results. In many experiments, the final classification accuracy of the test set is obtained through trial and error for each parameter.  Table 3 lists the optimal network parameters for the maximum classification accuracy of the algorithm model.

**Figure 8:**
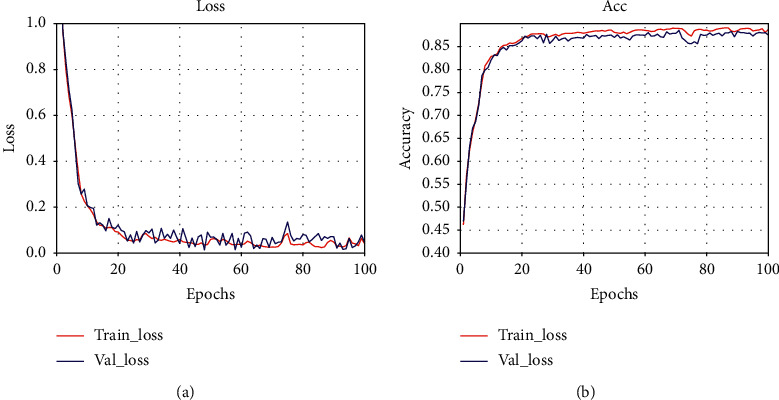
Training of the MLCNN-BiLSTM. (a) Loss curve. (b) Accuracy curve.

The experimental results show that, after 100 epochs of iterative training, the model converges and classification accuracy is stable. The results are shown in Figure 8.

Table 4 shows the ECG classification performance of different classes. In disease classification, the false negative (FN) results are very crucial. In a multiclass classification disease task, FN means that a patient has one disease, but the model predicts that he is healthy or has another disease. False negatives rate (FNR) can be shown by sensitivity (1 = FNR +SE). It can be seen from the sensitivity that, due to the imbalance in the number of samples of different disease, the proposed model has a good ability to identify normal, atrial arrhythmia, ventricular arrhythmia, and ventricular hypertrophy but not myocardial infarction and atrial hypertrophy.

In the experiment, we mainly study the influence of four different network learning parameters, namely, optimizer, batch size, convolution kernel size, and *α*, on the performance of the proposed model. When a certain parameter is changed, other parameters are consistent, as shown in Table 3.

First, the influences of different optimizers on the performance of the proposed network model are evaluated. The comparative experimental results are shown in Table 5. In terms of classification performance, the accuracy, sensitivity, and specificity of Adam are higher than those of SGD by 1.79%, 2.13%, and 2.18%, respectively.

Batch size represents the amount of data sent to the model for training each time, and its size affects degree of optimization and training speed of the model. Limited by the size of GPU memory used in this paper, we use the common parameters of 32, 64, 128, and 256 to carry out comparative experiments. The experimental results are shown in Table 6. The highest accuracy (87.81%) is obtained at a batch size of 128. The classification performance of our proposed network model is not significantly improved by changing the value of batch size.

Convolution kernel size is the smallest area where feature points are weighted and averaged in the convolution operation. The convolution kernel of the MLCNN proposed in this paper is the weighted average of a time series before and after any sampling point and related sampling points on different leads at the same time. We select four common convolution kernel sizes to explore their influence on model performance. Limited by the 12-lead ECG data structure adopted in this paper, the maximum convolution kernel is 9 × 9. The comparative experimental results are shown in Table 7. The convolution kernel with a size of 5 × 5 obtains an optimal result. The accuracy, sensitivity, and specificity are 87.81%, 86.00%, and 87.76%, respectively.

We study the influence of initializing the value of the threshold weight parameter *ɑ* on the performance of the proposed network model. The parameter *ɑ* controls the weight of the weighted fusion of the high-dimensional space-time features output by the two channels. The parameter *ɑ* indicates the sensitivity of different diseases to morphological and temporal features contained in ECG. After the backpropagation algorithm update, the parameter *ɑ* obtains the optimal value. The experimental results are shown in Table 8, and when the parameter *ɑ* is 0.7, the performance of the model is optimal. The experimental results show that the network model tends to use the spatial morphological features learned by the MLCNN channel as the main features after feature fusion for classification tasks.

Finally, to verify the effectiveness of our proposed ECG signal preprocessing method, the proposed network is also analyzed without denoising and the results are listed in Table 9. Average accuracy, sensitivity, and specificity of 85.53%, 84.71%, and 85.23% are achieved using raw ECG data with noise. Furthermore, the highest average accuracy of 87.81%, sensitivity of 86.00%, and specificity of 87.76% are obtained for preprocessed ECG data without noise.

## 5. Discussion

In this section, we construct CNN, BiLSTM, and MLCNN to carry out classification and recognition experiments on the same multilead ECG data set used in verifying the performance advantages of the proposed MLCNN in processing multilead ECG data with special structures. We also carry out experiments on the MLCNN-BiLSTM without the core threshold weight parameter *ɑ* to verify the necessity of introducing core threshold weight parameter *ɑ* for weighted fusion after the multichannel extraction of time-space features.

In the contrast experiment, the difference between CNN and MLCNN lies in whether the convolution kernel takes into account the four-neighborhood special structure of multilead ECG. CNN takes multilead ECG as a two-dimensional image and extracts signal features by traditional convolution. Our BiLSTM only uses standard II lead ECG as training data.

It can be seen from Table 10 that the classification performance of BiLSTM network is the worst, and ACC is only 67.70%. The possible reason is that the feature information in the data cannot be well expressed by using only a single two-layer LSTM network. Compared with the CNN, the performance of the MLCNN is further improved, with ACC, SE, and SP increased by 1.44%, 2.61%, and 3.93%, respectively. MLCNN is more suitable for multilead ECG data classification and recognition. The experimental results show that the MLCNN-BiLSTM proposed in this paper has the best performance among several models, its ACC is 87.81%, and it is effective in dealing with ECG classification.

Through feature visualization [6], the effectiveness of the model can be displayed intuitively. We select two cases of each different disease type from the test set to analyze the features learned from the MLCNN-BiLSTM model. Without using a softmax layer, we use the rest of the MLCNN-BiLSTM model as feature extractors, and the generated feature vector consists of 64 values. According to the learned feature vector, a heat map is drawn.

**Figure 9:**
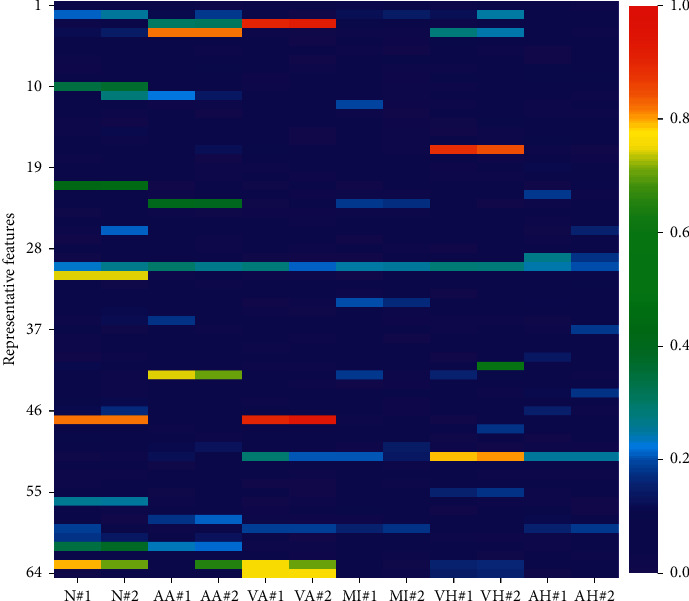
Visualization of the learned feature vectors. N# corresponds to the normal cases; AA# corresponds to the atrial arrhythmia cases patient; VA# corresponds to the ventricular arrhythmia cases patient; MI# corresponds to the myocardial infarction cases patient; VH# corresponds to the ventricular hypertrophy cases patient; AH# corresponds to the atrial hypertrophy cases patient.

As shown in Figure 9, through the color of features (10, 21, 31, 56), (4, 23, 42), (3, 47, 64), and (17, 51), we can clearly distinguish the cases of normal, atrial arrhythmia, ventricular arrhythmia, and ventricular hypertrophy from other types of cases. We notice that the visualization results of myocardial infarction and atrial hypertrophy are not ideal possibly because of the imbalance of training set samples and the failure of the MLCNN-BiLSTM model to learn key features from these two types of samples.

To evaluate the performance of our proposed network, we compare it with some of the latest methods in the literature.  Table 11 shows the application of the CCDD in ECG classification in the recent 5 years.

The literature studies [30–32] are based on the Lead Convolutional Neural Network (LCNN) [35]. The study in [30] proposed a novel supervised training method (i.e., implicit training method) and then combined it with a traditional training method (i.e., explicit training method), trained two different LCNN models, and finally used a Bayesian method to fuse classification. The study in [31] proposed an ensemble learning method based on LCNN and premature ventricular contraction diagnosis rules complementarily and achieved good classification results. The study in [32] adds LSTM on the basis of literature [31], and the classification performance was further improved. The study in [33] combined convolutional neural network and bidirectional recurrent neural network to establish a model for classifying ECG diseases as positive abnormalities. The study in [34] proposed an ensemble method based on a deep neural network, which integrated filtering view, local view, distortion view, explicit training, implicit training, subview prediction, and simple average, and verified the effectiveness of the method.

As shown in Table 11, the majority of ECG recognition and classification researches on the CCDD database had a binary classification. The study in [32] shows the performance in identifying PVC diseases. The results were better than those of the other binary classification studies and the accuracy rate reached 99.41%. For comparison with other advanced methods in the paper, we also conducted a binary classification study on the same data set using the proposed model. The accuracy, sensitivity, and specificity of the algorithm are 98.77%, 95.69%, and 97.35%, respectively, on the PVC disease data set with more than 140000 records, which verifies the effectiveness of the algorithm. Little research has been conducted on ECG multiclass classification recognition in the CCDD. We set up an MLCNN-BiLSTM parallel network. Through MLCNN and BiLSTM, two channels can fully extract the morphological features and rhythm features of multilead ECG. The results show that our work has good performance on the data set of the CCDD for classification and recognition of six diseases, and the accuracy rate is 87.81%.

## 6. Conclusion

In this study, we propose a neural network model named MLCNN-BiLSTM for cardiovascular disease recognition based on the special data structure of multilead ECG. The model is divided into two channels for the extraction of time-space feature information from ECG signal in parallel. The MLCNN channel sets the partial weights of the convolution kernel to 0, which can accurately extract the strongly correlated morphological features of the sampling points between different leads at the same time and the same lead at different times while ignoring irrelevant information. Compared with CNN, MLCNN decreases the tendency of overfitting during network memory consumption. The BiLSTM channel is used to extract rhythmic features. The core threshold parameters are initialized and the backpropagation algorithm is used to update automatically to fuse the spatiotemporal features extracted by multiple channels in parallel and for exploring the sensitivity of different cardiovascular diseases to morphological and rhythmic features. These networks are combined into a unified neural network architecture to form an end-to-end trainable model. The experimental results show that the accuracy of the MLCNN-BiLSTM is 87.81% in six cardiovascular disease classification tasks. It is 4.24% and 20.11% higher than the reference single CNN and BiLSTM, respectively.

The future work will be carried out in these aspects. First, we will further consider the impact of the imbalance on the performance of the network model. Second, given that multilead ECG data sets are few, we only carry out experiments on a single data set, and its generalization ability needs to be tested. In the next step, we will obtain a large amount of unlabeled data from the remote ECG monitoring system and continue to evaluate our proposed network model through unsupervised learning.

## Figures and Tables

**Algorithm 1:**
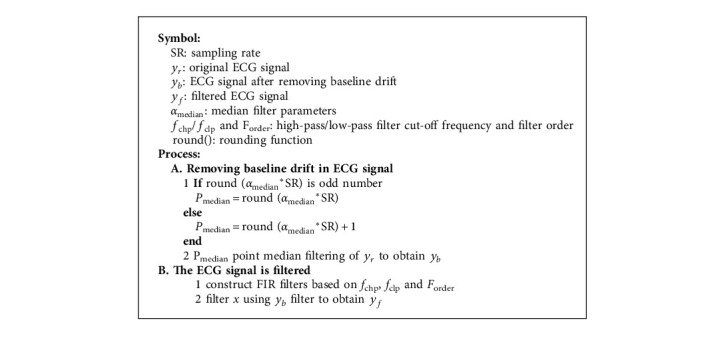
Preliminary processing of ECG.

**Algorithm 2:**
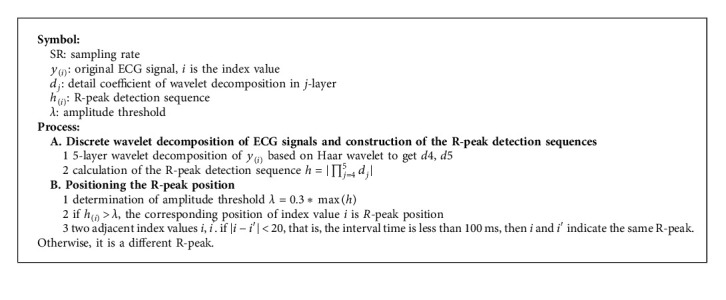
*R*-peak detection algorithm based on wavelet coefficients.

**Algorithm 3:**
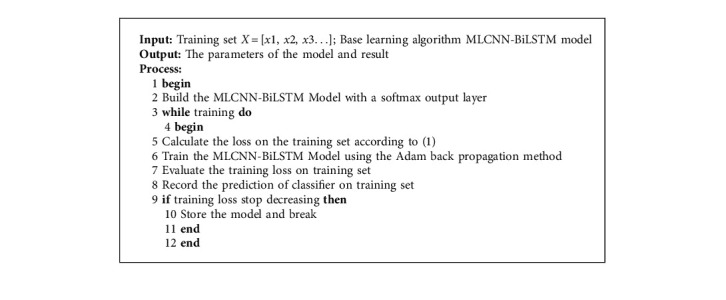
Training of MLCNN-BiLSTM model based classifier.

**Table 1:** Experimental data statistics.

Diseases	Train set	Validation set	Test set	Total

Normal	86496	10812	10812	108120
Atrial arrhythmia	12329	1541	1541	15411
Ventricular arrhythmia	4600	575	575	5750
Myocardial infarction	488	61	61	610
Ventricular hypertrophy	10468	1309	1309	13086
Atrial hypertrophy	91	12	12	115
Total	114472	14310	14310	143092

**Table 2:** Detailed parameters used for all the layers of proposed model.

Channel	Layer	Layer name	Kernel × unit	Other layer parameters

MLCNN	1	Conv2D	5 × 32	Strides = 1, padding = 2
	2	Batch norm.	—	—
	3	ReLU	—	—
	4	Max pooling 2D	2	Strides = 2
	5	Conv2D	5 × 64	Strides = 1, padding = 2
	6	Batch norm.	—	—
	7	ReLU	—	—
	8	Max pooling 2D	2	Strides = 2
	9	Conv2D	5 × 128	Strides = 1, padding = 2
	10	Batch norm.	—	—
	11	ReLU	—	—
	12	Max pooling 2D	2	Strides = 2
	13	Linear	256	ReLU, dropout

BiLSTM	1	BiLSTM	—	Hidden size = 128

—	1	Channel fusion	—	*α*
	2	Linear	512	ReLU, dropout
	3	Linear	128	ReLU, dropout
	4	Linear	64	ReLU, dropout

**Table 3:** Related parameter settings for the model proposed in this study.

Parameter	Value

Learning rate	0.001
Batch size	128
BiLSTM cell size	128
Convolution kernel size	(5,5)
Epochs	100
Optimizer	Adam
Cost function	Softmax
*α*	0.7

**Table 4:** Classification performance of different classes.

Diseases	TP	TN	FP	FN	ACC (%)	SE (%)	SP (%)

Normal	9897	2707	305	1401	88.08	87.60	89.87
Atrial arrhythmia	1076	11481	1551	202	87.75	84.19	88.10
Ventricular arrhythmia	530	12021	1659	100	87.71	84.13	87.87
Myocardial infarction	48	12451	1786	25	87.34	65.75	87.46
Ventricular hypertrophy	1007	11435	1553	315	86.95	76.17	88.04
Atrial hypertrophy	7	12447	1854	2	87.03	77.78	87.04

**Table 5:** Classification performance of different optimizer.

Optimizer	ACC (%)	SE (%)	SP (%)

Adam	87.81	86.00	87.76
SGD	86.02	83.87	85.48

**Table 6:** Classification performance of different batch size.

Batch Size	ACC (%)	SE (%)	SP (%)

32	77.75	73.48	75.60
64	87.43	85.58	85.42
128	87.81	86.00	87.76
256	87.53	82.48	84.33

**Table 7:** Classification performance of different convolution kernel size.

Convolution kernel size	ACC (%)	SE (%)	SP (%)

(3,3)	84.76	82.59	84.86
(5,5)	87.81	86.60	87.63
(7,7)	87.81	86.00	87.76
(9,9)	87.23	86.56	86.09

**Table 8:** Classification performance of different *ɑ*.

*α*	ACC (%)	SE (%)	SP (%)

0.4	81.30	77.64	73.36
0.5	85.71	80.77	82.95
0.6	87.07	86.25	86.96
0.7	87.81	86.00	87.76
0.8	86.94	85.15	85.54

**Table 9:** Classification performance of different convolution kernel size.

ECG data	ACC (%)	SE (%)	SP (%)

Raw ECG data	85.53	84.71	85.23
Preprocessed ECG data	87.81	86.00	87.76

**Table 10:** Comparison of experimental results with other models.

Model	ACC (%)	SE (%)	SP (%)

BiLSTM	67.70	74.36	76.34
CNN	83.57	79.32	81.89
MLCNN	85.01	81.93	85.82
MLCNN-BiLSTM (without *α*)	85.37	82.67	87.42
MLCNN-BiLSTM	87.81	86.00	87.76

**Table 11:** Summary of recent ECG classification methods on CCDD database.

Literature	Method	Classes	ACC (%)	SE (%)	SP (%)

Jin and Dong [30]	LCNN-A, LCNN-B, Bayesian	2	85.08	82.38	87.21
Zhou et al. [31]	LCNN, rules inference	2	97.87	87.94	98.02
Zhou et al. [32]	LCNN, LSTM, rules inference	2	99.41	97.59	99.54
Wang et al. [33]	CNN, BRNN	2	87.69	75.52	76.32
Jin and Dong [34]	DNN, ensemble learning	2	84.84	80.23	86.86
**Proposed**	**MLCNN, BiLSTM, feature fusion**	**2**	**98.77**	**95.69**	**97.35**
**Proposed**	**MLCNN, BiLSTM, feature fusion**	**6**	**87.81**	**86.00**	**87.76**

## Data Availability

The data used to support the findings of this study are included within the article. Further data can be requested from the corresponding author.
